# Evaluation of the 4Kscore Test in Relation to Subsequent Risk of Aggressive Prostate Cancer in the European Prospective Investigation into Cancer and Nutrition

**DOI:** 10.1158/1055-9965.EPI-24-1877

**Published:** 2025-08-22

**Authors:** K. Smith-Byrne, Georgina K. Fensom, Urwah Noor, Eleanor L. Watts, Naomi E. Allen, Pilar Amiano, María-Dolores Chirlaque, Marta Crous-Bou, Marcela Guevara Eslava, Domenico Palli, Verena A. Katzke, Carlotta Sacerdote, Maria-Jose Sánchez, Matthias B. Schulze, Sabina Sieri, Rosario Tumino, Konstantinos K. Tsilidis, Rudolf Kaaks, Marc J. Gunter, Elio Riboli, Timothy J. Key, Ruth C. Travis

**Affiliations:** 1 Cancer Epidemiology Unit, Nuffield Department of Population Health, University of Oxford, Oxford, United Kingdom.; 2 Big Data Institute, Nuffield Department of Population Health, University of Oxford, Oxford, United Kingdom.; 3 UK Biobank Ltd, Stockport, United Kingdom.; 4 Public Health Division of Gipuzkoa, BioDonostia Research Institute, Donostia San Sebastian, Spain.; 5 CIBER Epidemiology and Public Health (CIBERESP), Madrid, Spain.; 6 Department of Epidemiology, Regional Health Council, IMIB-Arrixaca, Murcia University, Murcia, Spain.; 7 Unit of Nutrition and Cancer, Cancer Epidemiology Research Program, Catalan Institute of Oncology (ICO), L'Hospitalet de Llobregat, Barcelona, Spain.; 8 Nutrition and Cancer Group, Bellvitge Biomedical Research Institute (IDIBELL), L'Hospitalet de Llobregat, Barcelona, Spain.; 9 Department of Epidemiology, Harvard T.H. Chan School of Public Health, Boston, Massachusetts.; 10 Navarra Public Health Institute, Pamplona, Spain.; 11 Navarra Institute for Health Research (IdiSNA), Pamplona, Spain.; 12 Cancer Risk Factors and Life-Style Epidemiology Unit, Institute for Cancer Research, Prevention and Clinical Network (ISPRO), Florence, Italy.; 13 Division of Cancer Epidemiology, German Cancer Research Center (DKFZ), Heidelberg, Germany.; 14 Unit of Cancer Epidemiology, Città della Salute e della Scienza University-Hospital, Turin, Italy.; 15 Escuela Andaluza de Salud Pública (EASP), Granada, Spain.; 16 Instituto de Investigación Biosanitaria ibs.GRANADA, Granada, Spain.; 17 Centro de Investigación Biomédica en Red de Epidemiología y Salud Pública (CIBERESP), Madrid, Spain.; 18 Department of Preventive Medicine and Public Health, University of Granada, Granada, Spain.; 19 Department of Molecular Epidemiology, German Institute of Human Nutrition Potsdam-Rehbruecke, Nuthetal, Germany.; 20 Institute of Nutrition Science, University of Potsdam, Nuthetal, Germany.; 21 Epidemiology and Prevention Unit, Fondazione IRCCS Istituto Nazionale dei Tumori di Milano, Milano, Italy.; 22 Cancer Registry and Histopathology Department, Provincial Health Authority (ASP), Ragusa, Italy.; 23 Department of Hygiene and Epidemiology, University of Ioannina School of Medicine, Ioannina, Greece.; 24 Department of Epidemiology and Biostatistics, School of Public Health, Imperial College London, London, United Kingdom.; 25 School of Public Health, Imperial College London, London, United Kingdom.

## Abstract

**Background:**

PSA is central to referrals for prostate biopsy but has low specificity for aggressive prostate cancer. This study evaluates the 4Kscore (OPKO Diagnostics) versus total PSA in predicting short- and long-term risks of aggressive prostate cancer.

**Methods:**

Baseline blood samples from 1,658 men diagnosed with prostate cancer (median diagnosis time = 8.6 years) and 1,658 matched controls in the European Prospective Investigation into Cancer and Nutrition were analyzed. Discrimination for the 4Kscore and total PSA was assessed using the AUC with 95% confidence intervals (CI) via bootstrapping.

**Results:**

For high-grade tumors, AUCs were 0.69 (95% CI, 0.66–0.72) for the 4Kscore and 0.75 (95% CI, 0.73–0.78) for total PSA. For advanced-stage disease, AUCs were 0.71 (95% CI, 0.66–0.75) for the 4Kscore and 0.77 (95% CI, 0.73–0.80) for total PSA. Similar findings were observed for other aggressive cancer endpoints. Among men with PSA >2 ng/mL, the 4Kscore had better discrimination than PSA for overall prostate cancer, high-grade disease, and prostate cancer death but only in men <60 years at recruitment.

**Conclusions:**

In this large European study, the 4Kscore did not significantly improve the prediction of clinically significant prostate cancer compared with total PSA, except in younger men with elevated PSA.

**Impact:**

The findings underscore the limited utility of the 4Kscore in improving medium- to longer-term risk prediction over PSA, with potential benefits restricted to younger men with elevated PSA.

## Introduction

Prostate cancer is the most commonly diagnosed cancer among men, with 1,467,854 cases diagnosed worldwide and 473,011 cases diagnosed in Europe annually (1). In current clinical practice, a patient’s circulating PSA concentration, digital rectal exam (DRE) results, age, family history, and ethnicity may all be considered when deciding whether to refer a man for a prostate biopsy. Although randomized trials have shown that PSA testing may reduce prostate cancer mortality (2, 3), there has been concern about the low specificity of the PSA test leading to overdiagnosis of nonaggressive disease and the potential clinical complications arising from prostate biopsies. The 4Kscore (OPKO Diagnostics) test was initially established to predict risk for high-grade prostate cancer in men with a moderately raised total PSA concentration (3–7 ng/mL). The 4Kscore test combines information from four kallikrein markers in the blood [total PSA, free PSA, intact PSA, and human kallikrein-related peptidase 2 (hK2), a serine protease coded for by a kallikrein gene and expressed primarily through the prostate epithelium (4, 5)], and age to provide patients with a predicted risk between 0% and 100% of them having a high-grade tumor (Gleason score ≥7) detected on a prostate biopsy (6).

Previous evidence has shown that PSA measures, including total and free PSA, as well as hK2, were significant predictors of prostate cancer diagnosed ≥20 years after blood collection (7, 8). However, there is limited evidence for the performance of the 4Kscore for prediction of medium- to long-term prostate cancer, as to date a substantial proportion of the research has focused on the short-term predictive accuracy of the 4Kscore to discriminate between patients with and without high-grade prostate cancer. For example, a previous meta-analysis that assessed the performance of the 4Kscore using data from approximately 11,000 patients reported an area under the receiver operating curve (AUC) of 0.81 for predicting Gleason score ≥7 cancer, but results for specific PSA ranges were not cited (9).

There is limited research on the diagnostic improvement of the 4Kscore in comparison with a model including PSA and age alone. Two previous studies using data from the European Randomized Study of Screening for Prostate Cancer in Rotterdam and Göteborg showed an improvement in the AUC for a base model of total PSA and age compared with a model additionally including free PSA, intact PSA and hK2, from 0.64 to 0.76 and 0.68 to 0.83, respectively (10, 11). However, both of these analyses were limited to men with elevated total PSA.

The majority of the research to date has assessed the discrimination of the 4Kscore in men with elevated PSA levels (≥3 ng/mL) at recruitment, by ascertaining controls as men with elevated PSA but a negative biopsy, such as with ProtecT (12). There is more limited evidence for the performance of the 4Kscore in men with prospective PSA concentrations that have a range more closely aligned with a normal control population. Given the high cost of this test and its proposed use as a reflexive score for a common clinical assay, further research is needed in this area (13). We used data from 1,658 prostate cancer cases and 1,658 matched controls from the European Prospective Investigation into Cancer and Nutrition (EPIC) study. Our primary aim was to test whether the 4Kscore improved prediction for medium- to long-term prostate cancer outcomes in comparison with a model consisting of total PSA at recruitment and age at recruitment, with kallikrein measures from recruitment blood samples. Additionally, we aimed to test the discrimination of the 4Kscore in comparison with total PSA for short-term diagnosis of aggressive disease across a range of PSA levels and ages.

## Materials and Methods

### Study population

The EPIC study is a prospective cohort study including 521,000 participants, of which 153,400 were men, from 23 centers across 10 European countries (14). Participants were recruited largely between 1992 and 2000, and at each of the centers, detailed information was collected from lifestyle questionnaires, anthropometric measurements, and dietary questionnaires. Baseline blood samples were taken from 139,600 men. All participants provided written informed consent, and the study was approved by the ethical review boards of the International Agency for Research on Cancer and the participating institutions. The following analyses use data from Germany, Italy, the Netherlands, Spain, and the United Kingdom.

### Follow-up

Cancer incidence, tumor subtypes, and vital status were identified through record linkage to regional and national registries, with the exception of Germany, where a variety of methods were used, including health insurance records, cancer and pathology registries, and active follow-up through study participants and their next of kin.

Cases were men who were diagnosed with incident prostate cancer (defined as code C61 in the 10th revision of the International Statistical Classification of Diseases and Related Health Problems) after blood collection and before the end of follow-up (December 2007–May 2010). An incidence density sampling procedure was used to match each case to a control participant at random, from a cohort of men who were alive and free of cancer (excluding nonmelanoma skin cancer, 10th revision of the International Statistical Classification of Diseases and Related Health Problems, C44) at the time of diagnosis. Cases were matched to controls based on recruitment center, length of follow-up (±6 months), age at blood collection (±6 months), time of blood collection (±1 hour), and fasting status at blood collection (<3, 3–6, >6 hours).

Tumor stage and grade were classified using the tumor–node–metastasis (TNM) system, histologic grade, and Gleason score. Tumor subgroups were defined as localized (≤T_2_ and N_0/x_ and M_0_, or stage coded as localized), advanced (T_3–4_ and/or N_1–3_ and/or M_1_, or coded as advanced), nonaggressive (≤T_3_ and N_0/x_ and M_0_), aggressive (a subset of advanced-stage disease defined as T_4_ and/or N_1–3_ and/or M_1_), low-grade (Gleason score <7 or coded as well or moderately differentiated tumors), and high-grade (Gleason score ≥7 or coded as undifferentiated or poorly differentiated tumors)_._ Information on tumor stage and grade at diagnosis was available for 1,034 (62%) and 1,397 (84%) cases, respectively. Additionally, during follow-up, 148 cases had prostate cancer listed as the underlying cause of death on their death certificate.

### Assessment of analytes

Analyses of total PSA, free PSA, intact PSA, and hK2 were conducted at the Wallenberg research laboratories, Department of Translational Medicine, Lund University, Skåne University Hospital, Malmö, Sweden, in citrated plasma samples. The dual-label DELFIA Prostatus total/free PSA Assay (Perkin Elmer) was used to measure total and free PSA (15). The performance of the Prostatus assay has been documented previously (16). The methodology and performance of the intact PSA assay (17–19) and the hK2 in-house research assay (7, 20–22) have been previously published. All assays were conducted with blinding to case or control status, and case–control sets were arranged in the same assay batch.

We note that in a pilot study, the concordance between measurements of analyte concentrations in serum and citrated plasma samples was assessed (*N* = 25 for serum and plasma, respectively), and a high concordance was observed (*r* = 0.98, 0.99, 0.74, and 0.98 for total PSA, free PSA, intact PSA, and hK2, respectively).

### Statistical analysis

Participants with concentrations of any analyte below the limit of detection (total PSA < 0.1, free PSA < 0.04, intact PSA < 0.01, and hK2 < 0.004; *N* = 32) along with the corresponding participant in their matched set (*N* = 32) were excluded from the analysis. Pearson’s *χ*^2^ tests and ANOVA were used to compare participant and prostate-specific biomarker characteristics between cases and controls.

For cross-sectional analyses, log transformations were applied to kallikrein protein concentrations and geometric means, adjusted for age at recruitment (<50, 50 to <55, 55 to <60, 60 to <65, 65 to <70, and ≥70 years), body mass index (continuous), study center, and batch, which were calculated in controls across a number of lifestyle characteristics. Pairwise correlations were calculated between the four prostate-specific biomarkers (total PSA, free PSA, intact PSA, and hK2) and age in relation to the 4Kscore for cases and controls combined.

The discrimination of a model of 4Kscore and a model of total PSA, both adjusted for age at recruitment (continuous), was assessed using the AUC, and 95% confidence intervals (CI) were calculated using bootstrapping with 1,000 replications. Discrimination was calculated for overall prostate cancer, high-grade tumors, advanced-stage tumors, aggressive disease, and death from prostate cancer, and for each outcome, additional analyses in a restricted subset of participants with total PSA ≥1 ng/mL, total PSA ≥2 ng/mL, total PSA ≥3 ng/mL, and age ≥60 years were also completed. For subgroup analyses, separate case–control matching was conducted for the following endpoints, namely high-grade tumor, advanced stage, aggressive disease, and prostate cancer death, enabling the selected controls to represent a reference population of both healthy men and men diagnosed with low-grade or localized disease. For the latter endpoints, one participant was randomly selected as a control for each case and matched on study center, age at recruitment (±12 months), and end of follow-up, meaning that controls had to be alive and free from aggressive, high-grade, or advanced disease, depending on the endpoint of interest, up until the case’s date of diagnosis (23).

The comparative discrimination of the 4Kscore test and the total PSA model was assessed by calculating the difference in AUC for each pair of models, and 95% CIs were calculated using bootstrapping with 1,000 replications. Bootstrapping was not performed where comparisons had fewer than 10 cases and 10 controls.

All data preparation and statistical analyses were run in duplicate by two independent analysts. All statistical tests are two-sided and were conducted using STATA software version 15 (College Station, TX: StataCorp LP, RRID: SCR_012763).

## Results

Data from 1,658 men diagnosed with prostate cancer and 1,658 matched controls were included in the analyses. The median age at blood collection was 59 years (IQR, 54–63 years), and, for cases, the median time between blood collection at recruitment and diagnosis was 8.6 years (IQR, 6.3–10.5 years). No significant differences were observed for selected baseline characteristics between cases and controls (Table 1). Median concentrations of all analytes at blood collection were higher in cases compared with controls; total PSA (median in cases = 2.25 ng/mL and median in controls = 0.81 ng/mL), intact PSA (cases = 0.30 ng/mL and controls = 0.16 ng/mL), free PSA (cases = 0.47 ng/mL and controls = 0.27 ng/mL), and hK2 (cases = 0.05 ng/mL and controls = 0.03 ng/mL). Additionally, the median 4Kscore was higher in cases than in controls (median in cases = 6% and median in controls = 4%; Table 1).

**Table 1.** Characteristics of control participants and patients with prostate cancer.

Characteristic	Controls (*n* = 1,658)	Cases (*n* = 1,658)	*P*
Age at blood collection, years	58.2 (6.8)	58.2 (6.8)	0.97
Weight, kg	80.1 (11.4)	80.2 (11.5)	0.81
Height, cm	173.0 (7.1)	172.7 (7.0)	0.19
Body mass index, kg/m^2^	26.7 (3.4)	26.9 (3.4)	0.27
Smoking status, *n* (%)	​	​	​
Never	505 (30.5)	563 (34.0)	​
Previous	742 (44.8)	716 (43.2)	​
Current	386 (23.3)	351 (21.2)	0.14
Alcohol, *n* (%)	​	​	​
<8	573 (34.6)	571 (34.4)	​
8–15	336 (20.3)	334 (20.1)	​
16–39	467 (28.2)	448 (27.0)	​
>40	282 (17.0)	305 (18.4)	0.73
Physical activity, *n* (%)	​	​	​
Inactive	244 (14.7)	238 (14.4)	​
Moderately inactive	466 (28.1)	468 (28.2)	​
Active	913 (55.1)	910 (54.9)	0.87
Educational attainment, *n* (%)	​	​	​
Primary/none	586 (35.3)	593 (35.8)	​
Tech	416 (25.1)	389 (23.5)	​
Secondary	167 (10.1)	161 (9.7)	​
Degree	423 (25.5)	431 (26.0)	0.51
Median analyte concentration at blood collection	​	​	​
Total PSA, ng/mL	0.81	2.25	​
Intact PSA, ng/mL	0.16	0.30	​
Free PSA, ng/mL	0.27	0.47	​
hK2, ng/mL	0.03	0.05	​
4K Score, %	3.83	6.46	​
Time to diagnosis, *n* (%)	​	​	​
<2 years	​	68 (4.1)	​
2 to <4 years	​	92 (5.6)	​
4 to <6 years	​	212 (12.9)	​
6 to <8 years	​	324 (19.7)	​
8 to <10 years	​	952 (57.8)	​
Year of diagnosis, median (range)	​	2004 (1994–2009)	​
Age at diagnosis, years (SD)	​	66 (6.8)	​
Tumor stage	​	​	​
Localized	​	727 (43.8)	​
Advanced	​	307 (18.5)	​
Gleason grade	​	​	​
≤6	​	534 (32.2)	​
7	​	402 (24.2)	​
≥8	​	180 (10.9)	​
Tumor grade	​	​	​
Low grade	​	753 (53.9)	​
High grade	​	644 (46.1)	​
PSA (ng/mL) at diagnosis	​	​	​
<3	​	17 (1.0)	​
≥3 and <10	​	307 (18.5)	​
≥10 and <40	​	161 (9.7)	​
≥40	​	40 (2.4)	​
Death from prostate cancer, *n*	​	148	​

In controls, the mean total PSA was higher in men who were older at blood collection and who were not diabetic (Table 2). The 4Kscore was 5-fold higher in men ages 70 to 74 years compared with men ages 45 to 49 years (mean 4Kscore = 9.5; 95% CI, 8.6–10.5 and 1.9; 1.8–2.1, respectively).

**Table 2.** Adjusted geometric mean PSA concentration (ng/mL) and 4Kscore in controls by selected characteristics.

​	Total PSA, ng/mL			4Kscore, %		
Factor and subset	*N*	Mean (95% CI)^a^	*P* difference	*N*	Mean (95% CI)^a^	*P* difference
Age at recruitment	​	​	​	​	​	​
45–49	179	0.606 (0.545–0.674)	​	179	0.019 (0.018–0.021)	​
50–54	292	0.708 (0.651–0.769)	​	292	0.029 (0.028–0.031)	​
55–59	464	0.817 (0.765–0.872)	​	464	0.038 (0.036–0.039)	​
60–64	491	0.986 (0.924–1.051)	​	491	0.051 (0.049–0.053)	​
65–69	143	1.389 (1.234–1.564)	​	143	0.061 (0.057–0.066)	​
70–74	89	1.863 (1.601–2.167)	<0.0001	89	0.095 (0.086–0.105)	<0.0001
Weight (kg)	​	​	​	​	​	​
≤60	42	0.785 (0.630–0.978)	​	42	0.040 (0.035–0.047)	​
>60–70	253	0.869 (0.794–0.950)	​	253	0.040 (0.038–0.043)	​
>70–80	610	0.943 (0.890–0.999)	​	610	0.042 (0.040–0.043)	​
>80–90	472	0.883 (0.827–0.942)	​	472	0.039 (0.038–0.041)	​
>90–100	194	0.845 (0.763–0.936)	​	194	0.038 (0.035–0.040)	​
>100	87	0.829 (0.712–0.966)	0.1997	87	0.039 (0.035–0.043)	0.1979
Height (cm)	​	​	​	​	​	​
≤160	62	0.892 (0.743–1.070)	​	62	0.040 (0.036–0.046)	​
>160–170	488	0.872 (0.817–0.931)	​	488	0.041 (0.039–0.043)	​
>170–180	861	0.899 (0.856–0.943)	​	861	0.040 (0.039–0.041)	​
>180–190	235	0.916 (0.834–1.006)	​	235	0.039 (0.037–0.042)	​
>190	12	0.795 (0.527–1.198)	0.8905	12	0.037 (0.028–0.048)	0.7304
Body mass index	​	​	​	​	​	​
<20	514	0.920 (0.864–0.979)	​	514	0.040 (0.039–0.042)	​
25–29	881	0.900 (0.858–0.944)	​	881	0.040 (0.039–0.042)	​
30+	263	0.816 (0.748–0.891)	0.0834	263	0.039 (0.036–0.041)	0.3473
Smoking status	​	​	​	​	​	​
Never	505	0.919 (0.862–0.979)	​	505	0.040 (0.039–0.042)	​
Previous	742	0.866 (0.822–0.913)	​	742	0.039 (0.038–0.041)	​
Current	386	0.910 (0.846–0.979)	0.3204	386	0.041 (0.039–0.043)	0.4491
Alcohol (grams/day)	​	​	​	​	​	​
<8	573	0.919 (0.866–0.976)	​	573	0.040 (0.039–0.042)	​
8–15	336	0.920 (0.851–0.994)	​	336	0.040 (0.038–0.042)	​
16–39	467	0.874 (0.818–0.933)	​	467	0.040 (0.039–0.042)	​
>40	282	0.838 (0.770–0.912)	0.3204	282	0.039 (0.037–0.042)	0.8539
Physical activity	​	​	​	​	​	​
Inactive	244	0.911 (0.831–1.000)	​	244	0.039 (0.037–0.042)	​
Moderately inactive	466	0.893 (0.836–0.953)	​	466	0.040 (0.039–0.042)	​
Active	913	0.885 (0.844–0.927)	0.8556	913	0.040 (0.039–0.041)	0.7496
Education level	​	​	​	​	​	​
Primary	586	0.832 (0.783–0.883)	​	586	0.039 (0.037–0.040)	​
Secondary	416	0.915 (0.854–0.981)	​	416	0.042 (0.040–0.044)	​
Tech	167	0.832 (0.744–0.931)	​	167	0.040 (0.037–0.043)	​
Degree	423	0.922 (0.858–0.990)	0.0831	423	0.038 (0.037–0.040)	0.0421
Diabetic	​	​	​	​	​	​
No	1,569	0.900 (0.868–0.933)	​	1,569	0.040 (0.039–0.041)	​
Yes	84	0.749 (0.641–0.876)	0.0253	84	0.040 (0.036–0.044)	0.9112

a Geometric means adjusted for age at blood collection, body mass index, center, and batch.

The four prostate-specific biomarkers that are incorporated in the 4Kscore were all significantly positively correlated with the 4Kscore, with the strongest correlation seen for total PSA (range for pairwise correlation *r* = 0.54–0.82, *P* < 0.0001 for all) when combining data for both cases and controls (Table 3). When the dataset was restricted to cases and controls with total PSA ≥2 ng/mL, the strong positive correlation between total PSA and the 4Kscore remained (*r* = 0.78, *P* < 0.0001).

**Table 3.** Correlations between analytes and 4Kscore.

​	Rho	*P* value
Total PSA	0.8230	<0.0001
Total PSA ≥2	0.7798	<0.0001
Free PSA	0.5406	<0.0001
Intact PSA	0.6000	<0.0001
hK2	0.5976	<0.0001
Age at blood collection	0.3150	<0.0001

**Figure 1.**
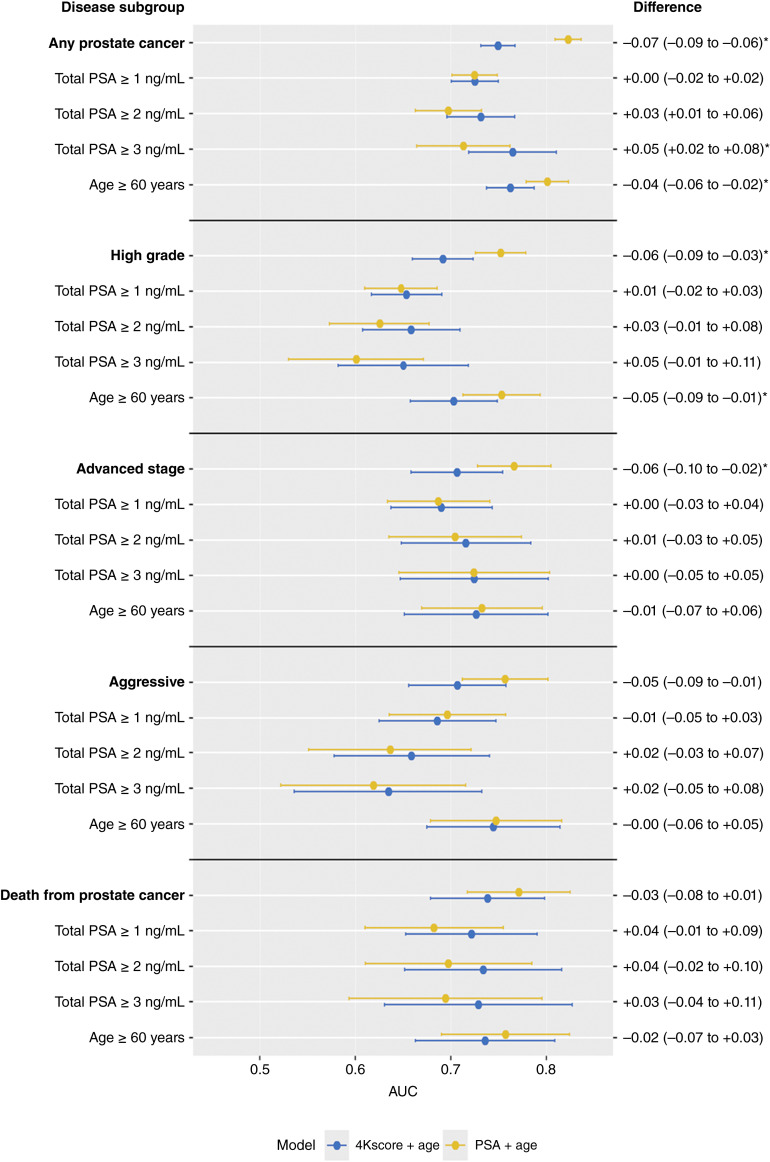
AUC results stratified by disease subgroup (overall prostate cancer, high-grade and advanced-stage tumors, and death from prostate cancer), PSA level at recruitment (all total PSA levels and ≥1, ≥2, and ≥3 ng/mL), and for men older than 60 years of age at recruitment. Yellow points refer to results from a total PSA + age at recruitment model, whereas blue points refer to results from a 4Kscore + age at recruitment model.

The AUCs for the 4Kscore model ranged from 0.63 to 0.76 across the predefined subgroups, with the highest discrimination for predicting overall risk of disease in participants with total PSA ≥3 ng/mL (Fig. 1; Table 4). The AUCs for the total PSA model ranged from 0.60 to 0.82.

**Table 4.** AUC results for PSA and the 4Kscore models with prostate cancer overall and clinically relevant disease subtypes and among men with elevated PSA who developed cancer.

​	Cases/controls	AUC PSA + age	AUC PSA + age SE	AUC 4Kscore + age	AUC 4Kscore + age SE	AUC difference (PSA + age vs. 4Kscore + age)	AUC difference SE (PSA + age vs. 4Kscore + age)	*P* value PSA + age vs. 4Kscore + age
Overall	1,658/1,658	0.82 (0.81–0.84)	0.0070	0.75 (0.73–0.77)	0.0091	−0.07 (−0.09, −0.06)	0.0081	**<0.0001**
PSA ≥1	1,420/652	0.73 (0.70–0.75)	0.0121	0.73 (0.70–0.75)	0.0125	0.00 (−0.02, 0.02)	0.0107	0.9792
PSA ≥2	921/258	0.70 (0.66–0.73)	0.0177	0.73 (0.70–0.77)	0.0181	0.03 (0.01, 0.06)	0.0147	**0.0214**
PSA ≥3	594/127	0.71 (0.66–0.76)	0.0249	0.76 (0.72–0.81)	0.0233	0.05 (0.02, 0.08)	0.0156	**0.0010**
Age ≥60	717/723	0.80 (0.78–0.82)	0.0113	0.76 (0.74–0.79)	0.0127	−0.04 (−0.06, −0.02)	0.0112	**0.0006**
High grade	626/626	0.75 (0.73–0.78)	0.0134	0.69 (0.66–0.72)	0.0162	−0.06 (−0.09, −0.03)	0.0132	**<0.0001**
PSA ≥1	539/319	0.65 (0.61–0.69)	0.0196	0.65 (0.62–0.69)	0.0190	0.01 (−0.02, 0.03)	0.0145	0.6830
PSA ≥2	337/149	0.63 (0.57–0.68)	0.0267	0.66 (0.61–0.71)	0.0262	0.03 (−0.01, 0.08)	0.0220	0.1286
PSA ≥3	230/77	0.60 (0.53–0.67)	0.0362	0.65 (0.58–0.72)	0.0349	0.05 (−0.01, 0.11)	0.0311	0.1114
Age ≥60	277/277	0.75 (0.71–0.79)	0.0206	0.70 (0.66–0.75)	0.0232	−0.05 (−0.09, −0.01)	0.0187	**0.0074**
Aggressive	201/201	0.76 (0.71–0.80)	0.0228	0.71 (0.66–0.76)	0.0259	−0.05 (−0.09, −0.01)	0.0224	**0.0258**
PSA ≥1	175/113	0.70 (0.64–0.76)	0.0313	0.69 (0.62–0.75)	0.0313	−0.01 (−0.05, 0.03)	0.0210	0.6207
PSA ≥2	128/52	0.64 (0.55–0.72)	0.0435	0.66 (0.58–0.74)	0.0417	0.02 (−0.03, 0.07)	0.0245	0.3470
PSA ≥3	95/34	0.62 (0.52–0.72)	0.0495	0.63 (0.54–0.73)	0.0502	0.02 (−0.05, 0.08)	0.0343	0.6520
Age ≥60 years	92/92	0.75 (0.68–0.82)	0.0350	0.74 (0.68–0.81)	0.0355	−0.00 (−0.06, 0.05)	0.0290	0.9204
Advanced	301/301	0.77 (0.73–0.80)	0.0196	0.71 (0.66–0.75)	0.0245	−0.06 (−0.10, −0.02)	0.0199	**0.0026**
PSA ≥1	261/148	0.69 (0.63–0.74)	0.0275	0.69 (0.64–0.74)	0.0273	0.00 (−0.03, 0.04)	0.0189	0.8686
PSA ≥2	175/74	0.70 (0.63–0.77)	0.0355	0.72 (0.65–0.78)	0.0348	0.01 (−0.03, 0.05)	0.0204	0.5787
PSA ≥3	130/50	0.72 (0.65–0.80)	0.0403	0.72 (0.65–0.80)	0.0397	0.00 (−0.05, 0.05)	0.0260	0.9953
Age ≥60 years	121/121	0.73 (0.67–0.80)	0.0322	0.73 (0.65–0.80)	0.0383	−0.01 (−0.07, 0.06)	0.0344	0.8567
Death from prostate cancer	143/143	0.77 (0.72–0.82)	0.0273	0.74 (0.68–0.80)	0.0305	−0.03 (−0.08, 0.01)	0.0219	0.1380
PSA ≥1	128/76	0.68 (0.61–0.76)	0.0371	0.72 (0.65–0.79)	0.0351	0.04 (−0.01, 0.09)	0.0249	0.1143
PSA ≥2	99/48	0.70 (0.61–0.78)	0.0446	0.73 (0.65–0.82)	0.0418	0.04 (−0.02, 0.10)	0.0302	0.2252
PSA ≥3	77/31	0.69 (0.59–0.80)	0.0516	0.73 (0.63–0.83)	0.0501	0.03 (−0.04, 0.11)	0.0377	0.3621
Age ≥60 years	94/94	0.76 (0.69–0.82)	0.0342	0.74 (0.66–0.81)	0.0372	−0.02 (−0.07, 0.03)	0.0267	0.4251

The 4Kscore did not significantly improve discrimination compared with total PSA for any of the main AUC analyses for estimating risk for subsequent diagnoses of overall prostate cancer or disease subtypes, including high-grade and advanced-stage tumors and aggressive and fatal disease. Specifically, the 4Kscore had significantly lower discrimination than total PSA for overall prostate cancer (AUCs of 0.75, 95% CI, 0.73–0.77 and 0.82, 0.81–0.84, respectively, AUC difference = 0.07, 95% CI, 0.06–0.09), high-grade tumors (0.69, 95% CI, 0.66–0.72 and 0.75, 0.73–0.78, respectively, AUC difference = 0.06, 95% CI, 0.03–0.09), aggressive disease (0.71, 95% CI, 0.66–0.76 and 0.76, 0.71–0.80, respectively, AUC difference = 0.05, 95% CI, 0.01–0.09), and advanced-stage disease (0.71, 95% CI, 0.66–0.75 and 0.77, 0.73–0.80, respectively, AUC difference = 0.06, 95% CI, 0.02–0.10; Fig. 1; Table 4). However, for analyses stratified by total PSA level, the 4Kscore significantly improved discrimination compared with total PSA for overall prostate cancer among men with total PSA ≥2 ng/mL (AUCs of 0.73, 95% CI, 0.70–0.77 and 0.70, 0.66–0.73, respectively, AUC difference = 0.03, 95% CI, 0.01–0.06) and total PSA ≥3 ng/mL (0.76, 95% CI, 0.72–0.81 and 0.71, 0.66–0.76, respectively, AUC difference = 0.05, 95% CI, 0.02–0.08) but not for any subtypes of aggressive prostate cancer (Fig. 1; Table 4).

**Figure 2.**
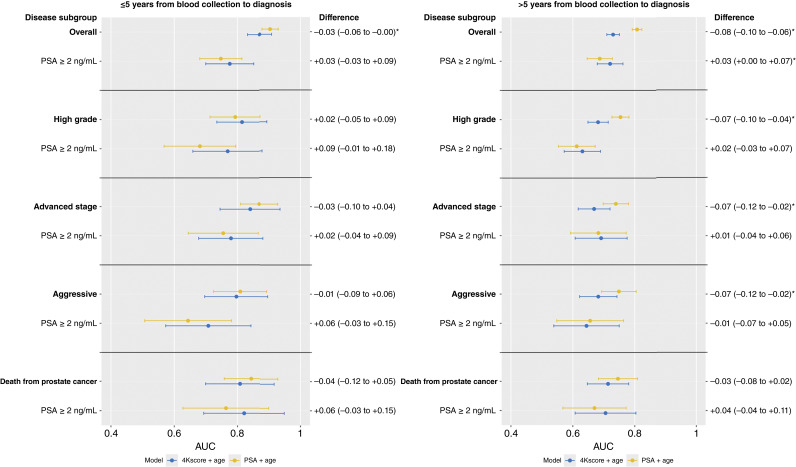
AUC results presented separately for cases diagnosed ≤5 years after blood collection and cases diagnosed >5 years after blood collection. The results are stratified by disease subgroup (overall prostate cancer, high-grade and advanced-stage tumors, and death from prostate cancer) and PSA level at recruitment (all total PSA levels and ≥2 ng/mL). Yellow points refer to results from a total PSA + age at recruitment model, whereas blue points refer to results from a 4Kscore + age at recruitment model.

**Figure 3.**
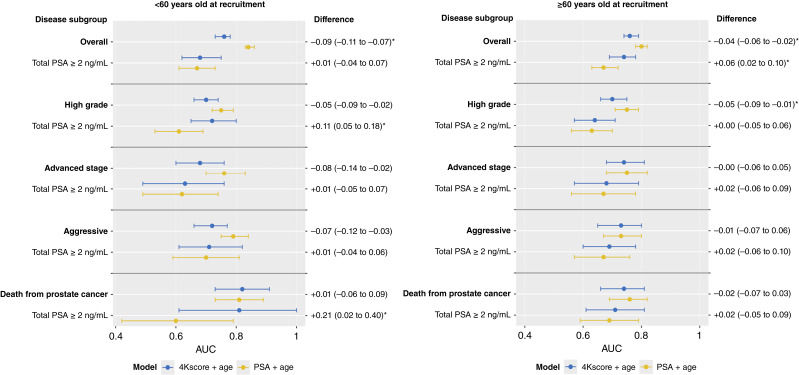
AUC results presented separately for cases recruited at < 60 years old and cases ≥60 years old at recruitment. The results are stratified by disease subgroup (overall prostate cancer, high-grade and advanced-stage tumors, and death from prostate cancer) and PSA level at recruitment (all total PSA levels and ≥2 ng/mL). Yellow points refer to results from a total PSA + age at recruitment model, whereas blue points refer to results from a 4Kscore + age at recruitment model.

Similar results were observed for analyses stratified by time between blood collection and diagnosis (Fig. 2; Supplementary Table S1; median lag time = 3.4 and 9.2 years for ≤5 years and >5 years, respectively). The results were materially similar in sensitivity analyses stratified by age at recruitment (Supplementary Table S2), country (Supplementary Table S3), and recruitment center (Supplementary Table S4), with two exceptions; an improvement in discrimination for the 4Kscore over total PSA was observed among men ages <60 years at recruitment with total PSA ≥2 ng/mL for both high-grade prostate cancer (0.72, 95% CI, 0.65–0.80 and 0.61, 0.53–0.69, respectively, AUC difference = 0.11, 95% CI, 0.05–0.18, Fig. 3) and death from prostate cancer (0.81, 95% CI, 0.61–1.00 and 0.60, 0.42–0.79, respectively, AUC difference = 0.21, 95% CI, 0.02–0.40, Fig. 3).

## Discussion

In this large European prospective study, with a median time between blood collection and diagnosis of 8.6 years, we compared the ability of the 4Kscore to improve prediction for medium- to long-term prostate cancer outcomes and short-term diagnosis of aggressive disease to a model of total PSA and age at recruitment. The 4Kscore did not significantly improve discrimination in main analyses, including by subtype of aggressive prostate cancer, for prostate cancer mortality or for prostate cancer in men with elevated PSA, with the exception of overall prostate cancer among men with elevated PSA. For high-grade prostate cancer, for example, the AUC for the 4Kscore was 0.69 compared with an AUC of 0.75 for total PSA, whereas in analyses restricted to men with total PSA ≥2 ng/mL, the corresponding AUCs were 0.66 and 0.63, respectively. However, we note that among men ages <60 years at recruitment and who also had elevated PSA, we saw some evidence that the 4K score improved discrimination for high-grade prostate cancer or death from prostate cancer.

The AUCs reported for the total PSA model in the current analyses are broadly similar to findings from previous studies (24). However, the AUCs reported for the 4Kscore are generally lower than those reported in previous studies, and our finding that the 4Kscore is not an improvement over total PSA for aggressive prostate cancer differs from the findings from some previous studies (24). There are several potential explanations for the differences between the current findings and previous studies.

First, our EPIC analyses explore how effective the 4Kscore is at predicting aggressive prostate cancer with a longer lag time between baseline blood collection and diagnosis (median of 8.6 years) than in previous studies. The OPKO 4Kscore test algorithm, which was used to generate the 4Kscore in the current evaluation, includes coefficients for the four prostate-specific biomarkers and age that were developed and evaluated in studies with a shorter follow-up (10–12, 25–29). However, when we performed subgroup analyses by time to diagnosis, whereas the AUCs for both total PSA and the 4Kscore were greater with shorter time to diagnosis, we did not find evidence for an improvement in performance of the 4Kscore relative to total PSA for discriminating aggressive prostate cancer overall.

Second, the range of PSA values in this prospective study is broader than in many previous studies that restricted their analyses to participants with elevated PSA concentrations (9). The current EPIC analyses are based on data from men with prospective total PSA values ranging from 0.2 to 43.2 ng/mL. However, when we restrict our analyses to participants with elevated PSA (≥2 and ≥3 ng/mL), we found that the 4Kscore model significantly improved discrimination compared with total PSA for overall prostate cancer, as well as separately for high-grade prostate cancer and prostate cancer death in men ages younger than 60 at recruitment.

Third, the EPIC study combines data from five European countries (Germany, Italy, the Netherlands, Spain, and the United Kingdom), whereas previous studies have been conducted in individual countries (France, the Netherlands, Spain, Sweden, the United Kingdom, and the United States; ref. 9). EPIC countries have diverse healthcare systems and guidelines for prostate biopsy referral that differ both from each other and from the US cohorts and European trials that reported an improvement in discrimination for the 4Kscore. Furthermore, differences by country in the age at diagnosis and the association of age at diagnosis with PSA may drive subtle differences in the predictive utility of a kallikrein risk-based algorithm. However, given that the results were qualitatively similar when analyses were stratified by country, this does not seem to explain our findings.

A recent study in the multiethnic cohort of 1,667 prostate cancer cases and 691 controls, which was more similar in design to the EPIC than some of the previous 4Kscore evaluation studies (in that both the EPIC and multiethnic cohort are prospective and with a wide range of PSA values at baseline; ref. 13), also reported no significant improvement in discrimination for the 4Kscore compared with a total PSA model for high-grade (Gleason score ≥8) or fatal prostate cancer in all men but did find an improvement for the 4Kscore compared with total PSA in analyses restricted to men with PSA >2 ng/mL. Further information on the performance of the 4Kscore could be produced by a randomized design, such as a trial on-going in Finland (30).

One limitation of our study is that the EPIC cohort does not include additional clinical parameters that are routinely combined with PSA and the 4Kscore—such as DRE findings and prior biopsy history. Consequently, our estimates of the discriminative performance of both PSA and the 4Kscore may not directly reflect those reported in studies in which such clinical details are available, and our findings may not fully replicate all aspects of its intended use. In clinical practice, the 4Kscore is applied as a secondary risk stratification tool, integrated with other clinical data to guide biopsy, MRI, genetic testing, and reflex testing decisions. We note that our measurements, taken at recruitment long before diagnosis, may underscore the potential utility of the 4Kscore at younger ages and believe that future studies with access to these clinical factors are needed. Such studies may be useful to inform the potential to extend the current use cases for the 4Kscore to men at younger ages.

We also acknowledge that we did not have information on why men were referred for biopsy. Information on whether referrals were indicated because of high PSA levels or an abnormal DRE would help differentiate men with a higher underlying risk or more aggressive disease from those referred for less specific reasons, which may have aided in the interpretation of the long-term discriminative value of the 4Kscore.

Using observational data from a large European prospective nested case–control study, we find that when estimating risk for subsequent diagnoses of prostate cancer subtypes of high-grade and advanced-stage tumors and aggressive and fatal disease, the 4K score test did not improve discrimination compared with a linear model of total PSA at recruitment. However, we find some evidence in the subgroup of men who were both younger than age 60 years at recruitment and who had an elevated blood PSA level for an improvement by the 4K score compared with PSA alone for the detection of clinically significant disease. If replicated, these findings may guide the future application of the 4Kscore, especially when integrated with emerging approaches such as polygenic risk scores and novel highly multiplex proteomics technologies, genetic adjustment of biomarkers, and the incorporation of rare variant data for improved risk prediction.

## Supplementary Material

Supplementary Table 1 AUC results for PSA and the 4K score models with prostate cancer overall and clinically relevant disease subtypes and among men with elevated PSA developed cancer within five years of recruitment and more than five years after recruitment

Supplementary Table 2 AUC results for PSA and the 4K score models with prostate cancer overall and clinically relevant disease subtypes and among men with elevated PSA who were under the age of 60 at recruitment and over the age of 60 at recruitment

Supplementary Table 3 AUC results for PSA and the 4K score models with prostate cancer overall and clinically relevant disease subtypes and among men with elevated PSA stratified by EPIC recruitment country

Supplementary Table 4 AUC results for PSA and the 4K score models with prostate cancer overall and clinically relevant disease subtypes stratified by EPIC recruitment centre

## Data Availability

Information on how to submit an application for gaining access to EPIC data is given at https://epic.iarc.fr/access/missionstatement.php
